# Combination of polyetherketoneketone scaffold and human mesenchymal stem cells from temporomandibular joint synovial fluid enhances bone regeneration

**DOI:** 10.1038/s41598-018-36778-2

**Published:** 2019-01-24

**Authors:** Yi Lin, Mayumi Umebayashi, Mohamed-Nur Abdallah, Guoying Dong, Michael G. Roskies, Yaoyao Fiona Zhao, Monzur Murshed, Zhiguang Zhang, Simon D. Tran

**Affiliations:** 1 Department of Oral and Maxillofacial Surgery, Guanghua School of Stomatology, Hospital of Stomatology, Sun Yat-sen University, Guangdong Provincial Key Laboratory of Stomatology, Guangzhou, P. R. China; 2 Craniofacial Tissue Engineering and Stem Cells Laboratory, Faculty of Dentistry, McGill University, Montreal, QC Canada; 3 Discipline of Orthodontics, Faculty of Dentistry, University of Toronto, Toronto, ON Canada; 4 Department of Mechanical Engineering, Faculty of Engineering, McGill University, Montreal, QC Canada; 5 Department of Otolaryngology-Head and Neck Surgery, McGill University, Montreal, QC Canada; 6 Division of Experimental Medicine, Department of Medicine, McGill University, Montreal, QC Canada; 7 Faculty of Dentistry, McGill University, Montreal, QC Canada; 8 Shriners Hospital for Children, McGill University, Montreal, QC Canada

## Abstract

Therapies using human mesenchymal stem cells (MSCs) combined with three-dimensional (3D) printed scaffolds are a promising strategy for bone grafting. But the harvest of MSCs still remains invasive for patients. Human synovial fluid MSCs (hSF-MSCs), which can be obtained by a minimally invasive needle-aspiration procedure, have been used for cartilage repair. However, little is known of hSF-MSCs in bone regeneration. Polyetherketoneketone (PEKK) is an attractive bone scaffold due to its mechanical properties comparable to bone. In this study, 3D-printed PEKK scaffolds were fabricated using laser sintering technique. hSF-MSCs were characterized and cultured on PEKK to evaluate their cell attachment, proliferation, and osteogenic potential. Rabbit calvarial critical-sized bone defects were created to test the bone regenerative effect of PEKK with hSF-MSCs. *In vitro* results showed that hSF-MSCs attached, proliferated, and were osteogenic on PEKK. *In vivo* results indicated that PEKK seeded with hSF-MSCs regenerated twice the amount of newly formed bone when compared to PEKK seeded with osteogenically-induced hSF-MSCs or PEKK scaffolds alone. These results suggested that there was no need to induce hSF-MSCs into osteoblasts prior to their transplantations *in vivo*. In conclusion, the combined use of PEKK and hSF-MSCs was effective in regenerating critical-sized bone defects.

## Introduction

Each year, there are 2.2 million bone grafting procedures performed worldwide in numerous medical disciplines including in orthopedics and in dentistry^1^. While autograft is still regarded as the gold standard of therapy, its disadvantages include donor site morbidity, prolonged recovery time, and contraindicated in certain populations (e.g. osteoporotic patients). Allogeneic bone grafts, on the other hand, possess the risk for an immunologic response or disease transfer^2–4^. Stem cell-based therapy for bone regeneration has been considered as the most promising approach to overcome the aforementioned limitations of bone grafting^5–8^. However, the optimal cell types and biomimetic scaffolds for bone regeneration remain to be explored.

Mesenchymal Stem Cells (MSCs), also recently defined as Medicinal Signaling Cells^9^, have been widely tested to provide grafts with intrinsic bone-forming capacity to regenerate substantial bone loss^10–12^. MSCs are present in various tissues and fluids, such as bone marrow^13,14^, adipose^15,16^, amniotic^17,18^, and synovial fluid^19,20^. Among these sources, human synovial fluid mesenchymal stem cells (hSF-MSCs) have drawn increasing interest to researchers because hSF-MSCs share certain similarities to bone marrow mesenchymal stem cells (BMSCs) in terms of their self-renewal, cell proliferation, and multilineage differentiation abilities^21^. More importantly, hSF-MSCs can be easily harvested from the patient’s joint lavage fluid by arthrocentesis, a minimally invasive procedure to aspirate/collect the joint fluid with a syringe, for diagnosis or treatment of articular cartilage damage^22,23^. hSF-MSCs can be found in various joint cavities and in a higher number in pathologic conditions^24–33^. Our previous studies indicated that hSF-MSCs could be collected from the synovial fluid of the temporomandibular joint (TMJ) in patients with temporomandibular disorders (TMD)^34^. hSF-MSCs were found to be multipotent, with a greater capacity for chondrogenesis; these cells were thus suggested to be best used for cartilage repair^24,26,35^. Although hSF-MSCs were also demonstrated to possess a greater osteogenic potential than MSCs from many other tissue sources^35^, their applications for bone regeneration have been barely investigated in the literature. This study aims to examine the bone regeneration capacity of hSF-MSCs.

Bone tissue engineering requires a three-dimensional (3D) biomaterial/scaffold to reconstruct osseous defects and to support the survival of the transplanted cells. Polyetherketoneketone (PEKK) has recently been demonstrated as an adequate 3D scaffold for bone tissue engineering due to its biocompatibility, biomechanical properties, and ease in prognostic evaluation (radiolucency) once transplanted *in vivo*^36–39^. The modulus of elasticity of PEKK (3–4 GPa) versus that in bone (18 GPa) resolves current stress shielding issues encountered in other categories of implants, such as metal grafts (100–210 GPa), that can lead to implant failure^38,40^. When compared to other polymers, PEKK has a higher strength which ensures structural rigidity^41^ for successful bone grafting. The radiolucency characteristic of PEKK^38,40,42^ also makes it easier to distinguish PEKK from newly-formed bone on radiographs. In addition, PEKK is a thermoplastic polymer, and thus can be 3D-printed with customized dimensions and shapes for patients^41–44^. PEKK has been FDA-approved in implant devices. However, one limitation of PEKK scaffold is its low osteoinductivity characteristic, but this can be enhanced by the incorporation of bioactive factors.

This study aimed to investigate further the *in vivo* osteogenic capability of hSF-MSCs when combined to 3D-printed PEKK scaffolds. We hypothesized that combining hSF-MSCs to PEKK scaffolds would enhance new bone formation *in vivo* in an established rabbit calvarial critical-sized defect (CSD). To the best of our knowledge, this is the first study in its kind.

## Results

### Characteristics of hSF-MSCs

**Figure 1:**
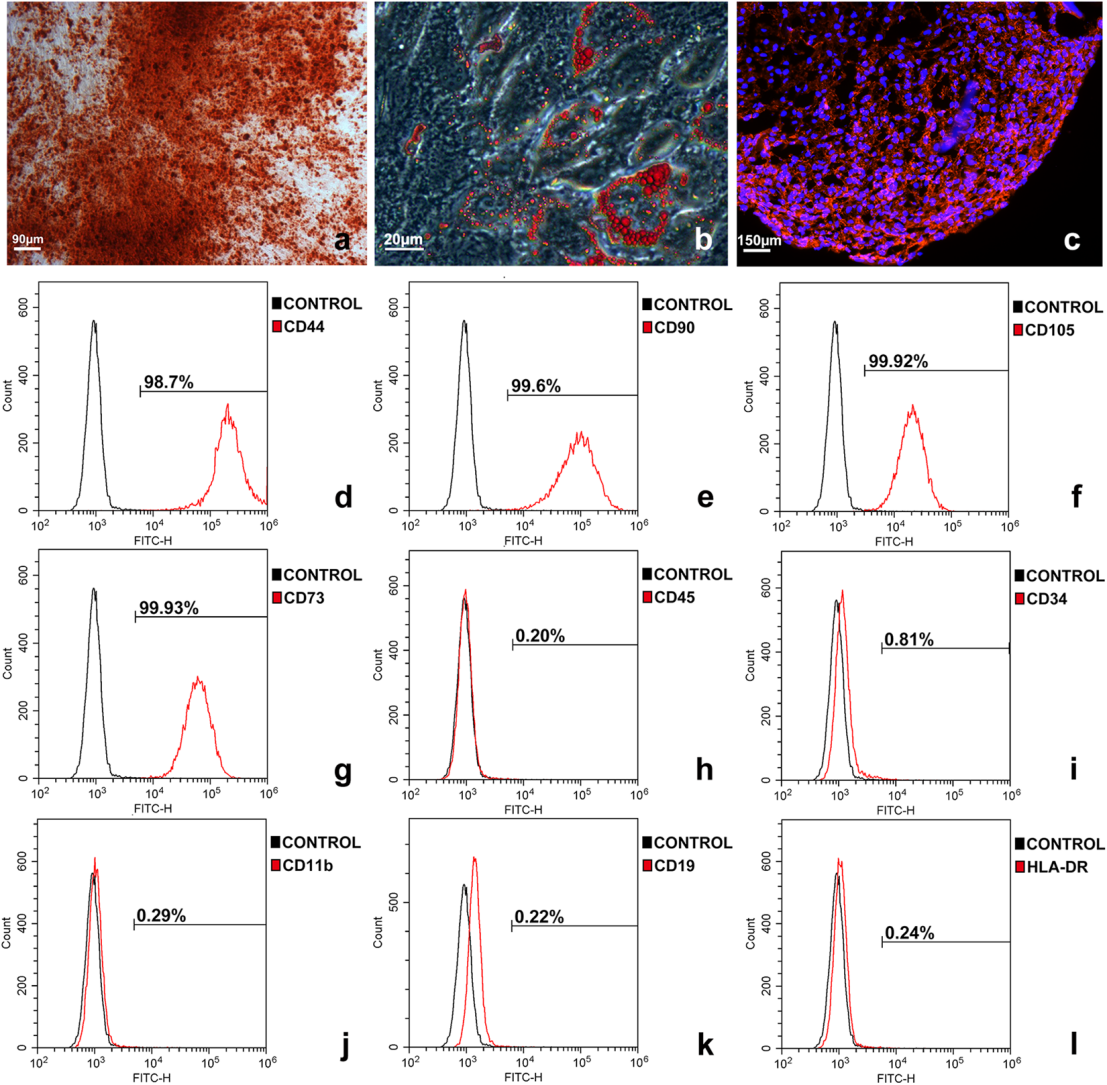
Characterization of hSF-MSCs. (**a**) Photomicrograph of calcified nodules stained by Alizarin Red indicating that hSF-MSCs had differentiated into an osteogenic cell lineage. (**b**) Oil Red O staining showing intracellular lipid droplets (red) in hSF-MSCs that were adipogenically-induced. (**c**) After chondrogenic differentiation of hSF-MSCs for 28 days, collagen type II was detected around cells by immunofluorescent staining. (**d–l**) Representative graphs of flow cytometry analysis of the phenotype of hSF-MSCs for MSC markers including CD44 (**d**), CD90 (**e**), CD 105 (**f**), and CD73 (**g**), and negative for CD45 (**h**), CD34 (**i**), CD11b (**j**), CD19 (**k**), and HLA-DR (**l**).

hSF-MSCs derived from five donors were used in this study. hSF-MSCs at cell passage 3 were assessed for their multilineage differentiation characteristics, according to the guidelines of the International Society of Cellular Therapy (ISCT). Osteogenic differentiation was demonstrated with calcium deposits (stained with Alizarin Red) after 21 days of culture (Fig. 1a). Adipogenic differentiation, after 14 days of culture, was detected with Oil Red O staining for cytoplasmic lipid granules (Fig. 1b). Chondrogenic differentiation was shown by positive immunofluorescent staining of collagen type II after 28 days of cell pellet culture in chondrogenic medium (Fig. 1c). Using flow cytometry (Fig. 1d–l), hSF-MSCs were confirmed for MSCs markers and expressed CD44 (99.46% ± 0.66), CD90 (98.89% ± 0.83), CD105 (97.38% ± 2.31), and CD73 (99.91% ± 0.06). hSF-MSCs were negative (0.12–0.58%) for CD45, CD34, CD11b, CD19, and HLA-DR.

### Attachment and proliferation of hSF-MSCs on PEKK scaffolds

**Figure 2:**
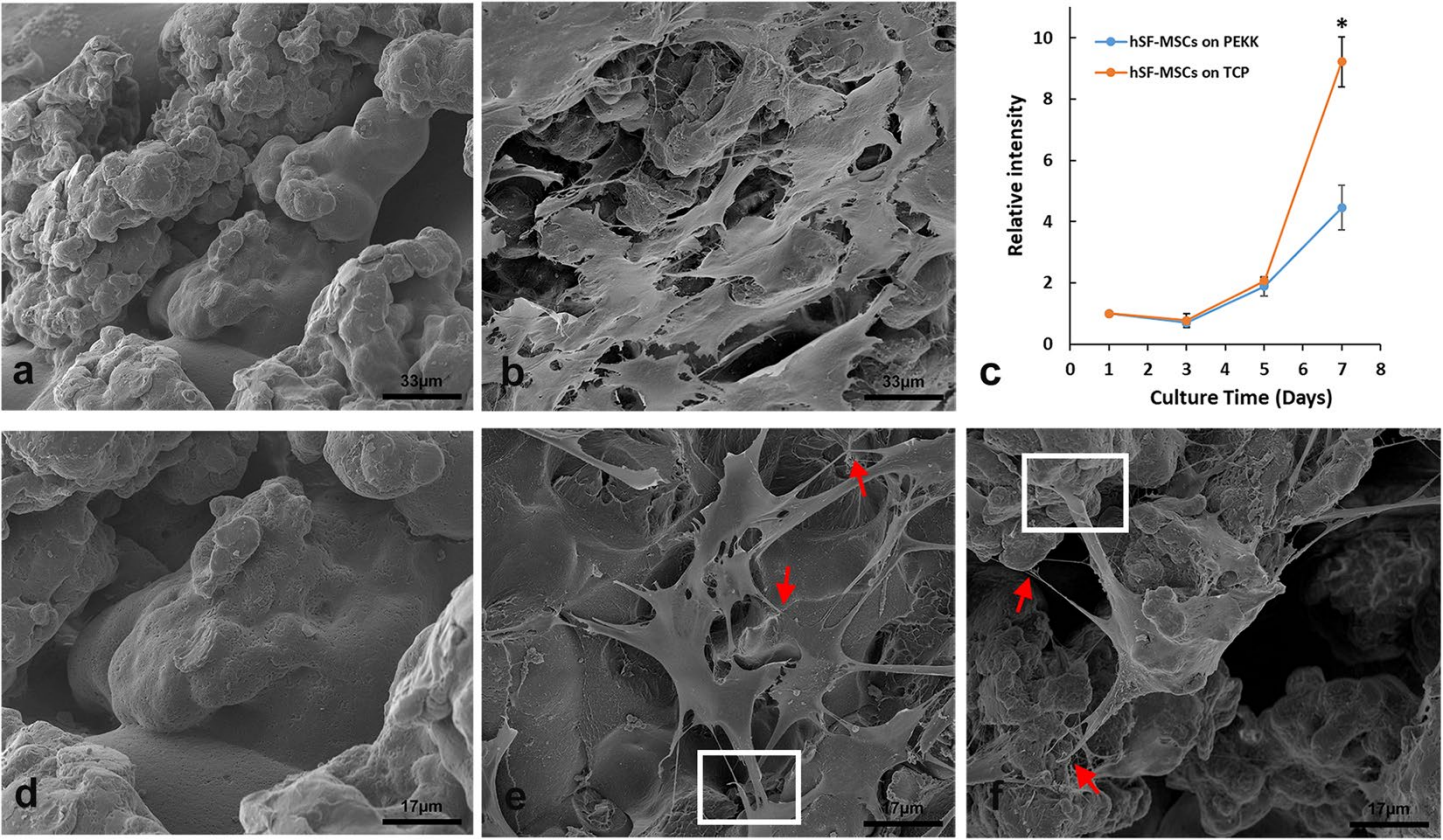
SEM morphology of 3D-printed PEKK and hSF-MSCs cultured on the surface of PEKK scaffolds. (**a**,**d**) The porous topography of PEKK scaffolds after 7 days of being immersed in culture media. (**b,e,f**) hSF-MSCs attached on PEKK scaffolds after 7-day incubation. Note: The red arrows indicate the filopodia, and the white rectangles indicate the lamellipodia of hSF-MSCs. (**c**) Cell growth curve of hSF-MSCs on PEKK versus TCP. Data are presented as mean ± SE. Differences were considered significant at *p < 0.05.

The biocompatibility of PEKK scaffolds cultured for 7 days with hSF-MSCs was evaluated by the cell attachment and cell growth assays. Scanning electron microscopy (SEM) showed PEKK exhibited a rough surface with opened micropores (Fig. 2a,d). The cell-seeded PEKK scaffold was attached with hSF-MSCs (Fig. 2b) showing cell membrane extensions such as filopodia and lamellipodia (Fig. 2e,f). Cell growth was measured by the Alamar blue assay. The proliferation rate of hSF-MSCs on PEKK and on tissue culture plastic (TCP) was similar at day 1, 3, and 5. However, the cell proliferation rate on TCP was twice that of PEKK on day 7 (Fig. 2c).

### Osteogenic characterisitic of hSF-MSCs on PEKK *in vitro*

**Figure 3:**
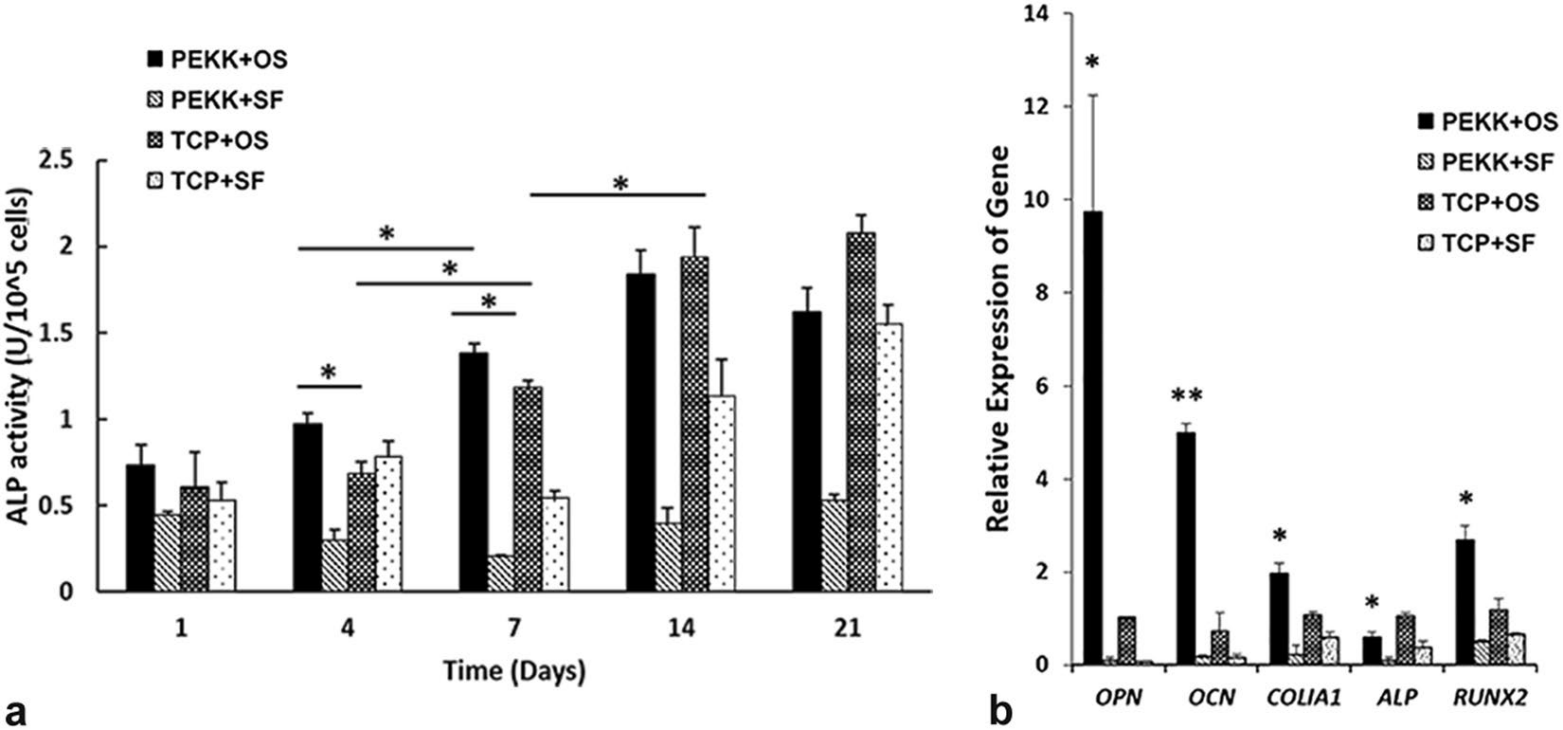
*In vitro* osteogenic ability of hSF-MSCs on PEKK scaffolds. (**a**) Standardized ALP activity of hSF-MSCs cultured on PEKK and TCP, with or without osteogenic induction for 1, 4, 7, 14, and 21 days. (**b**) *OPN*, *OCN*, *COLIA1*, *ALP*, and *RUNX2* gene expression of hSF-MSCs cultured for 21 days on PEKK or TCP, with or without osteogenic induction. Note: PEKK + SF: PEKK seeded with hSF-MSCs; PEKK + OS: PEKK seeded with osteogenically-induced hSF-MSCs; TCP + OS: TCP seeded with osteogenically-induced hSF-MSCs; TCP + SF: TCP seeded with hSF-MSCs. Data are presented as mean ± SE. *p < 0.05 and **p < 0.01.

The alkaline phosphatase (ALP) activity of hSF-MSCs seeded on PEKK scaffolds was higher than that on TCP, in osteogenic medium at day 4 and day 7 (*p* < 0.05) (Fig. 3a). At day 14 and 21, no statistically significant difference was found between the osteogenically-induced hSF-MSCs seeded on PEKK scaffolds (i.e. PEKK + OS group) versus osteogenically-induced hSF-MSCs cultured on plastic (TCP + OS group). Results of quantitative reverse transcription polymerase chain reaction (qRT-PCR) (Fig. 3b) demonstrated that osteogenically-induced hSF-MSCs on PEKK scaffolds (PEKK + OS) showed a significantly higher upregulation for *OPN* (9.6 folds), *OCN* (6.8 folds), *COLІA1* (1.8 folds), and *RUNX2* (2.2 folds) than osteogenically-induced hSF-MSCs cultured on plastic (TCP + OS) at day 21 of culture. Control groups (PEKK + SF and TCP + SF) were detected with negligible expression of the above-mentioned genes, and the differences between these two groups in ALP activity and gene expression were statistically not significant.

### *In vivo* evaluation of hSF-MSCs seeded on PEKK scaffolds

**Figure 4:**
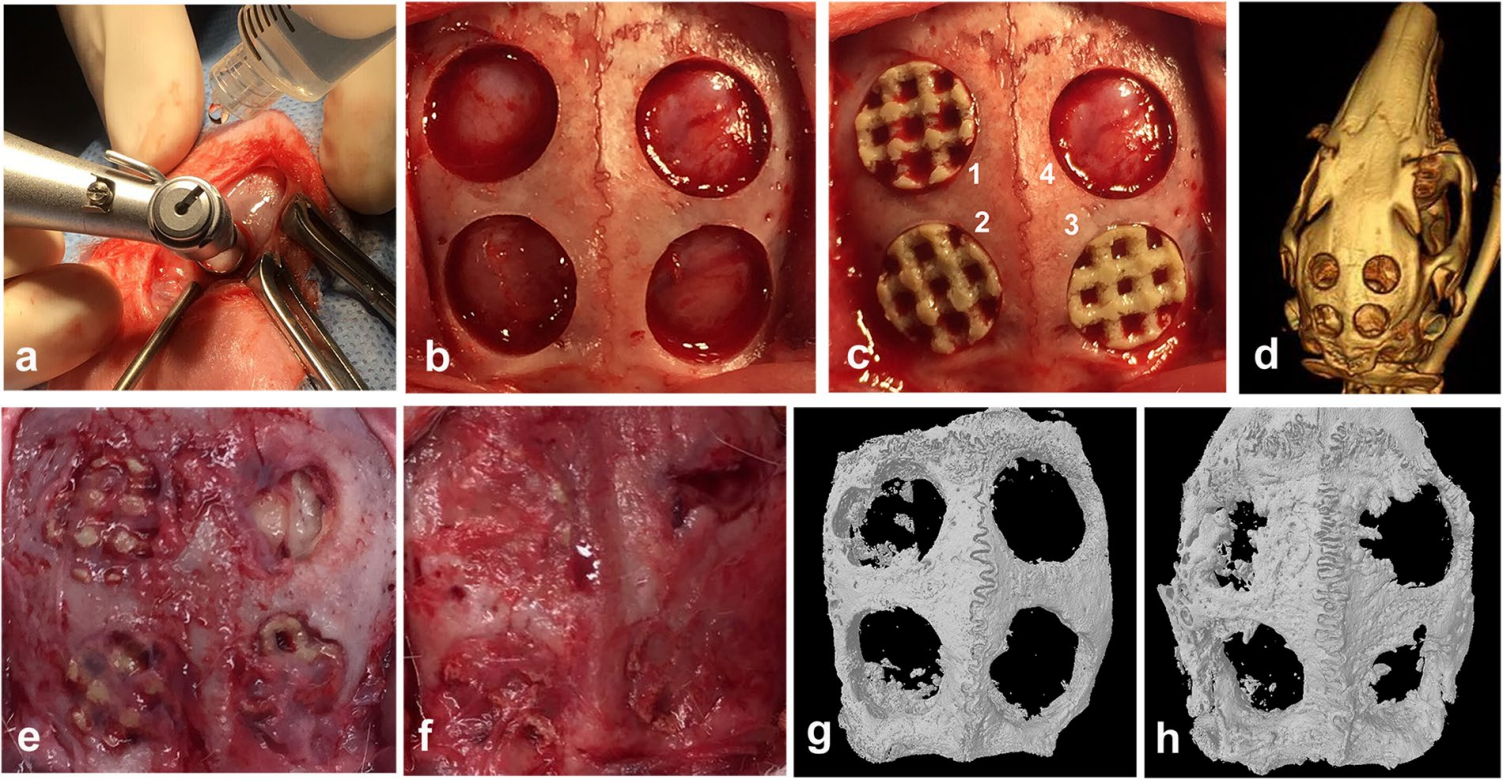
Rabbit critical-sized calvarial bone defect model. (**a**) The defects were created with a trephine bur (diameter, 8 mm) using continuous saline irrigation at the surgical site. (**b**) Four defects of 8mm-diameter were created on the parietal bone of rabbits. (**c**) Grafts were implanted randomly into the defects. (1) PEKK + SF (PEKK scaffold seeded with hSF-MSCs), (2) PEKK + OS (PEKK scaffold seeded with osteogenically-induced hSF-MSCs), (3) PEKK (scaffold only; no human cells), (4) Control (bone defect with no implant). (**d**) CT scan image of rabbit calvaria taken immediately after surgery. (**e,f**) Macroscopic appearance of the rabbit calvaria at 4 and 12 weeks post-surgery, respectively. (**g,h**) 3D reconstruction of micro-CT images of the rabbit calvaria explanted at 4 and 12 weeks post-surgery, respectively. The order and orientation of the implants were the same as the ones shown in panel c.

**Figure 5:**
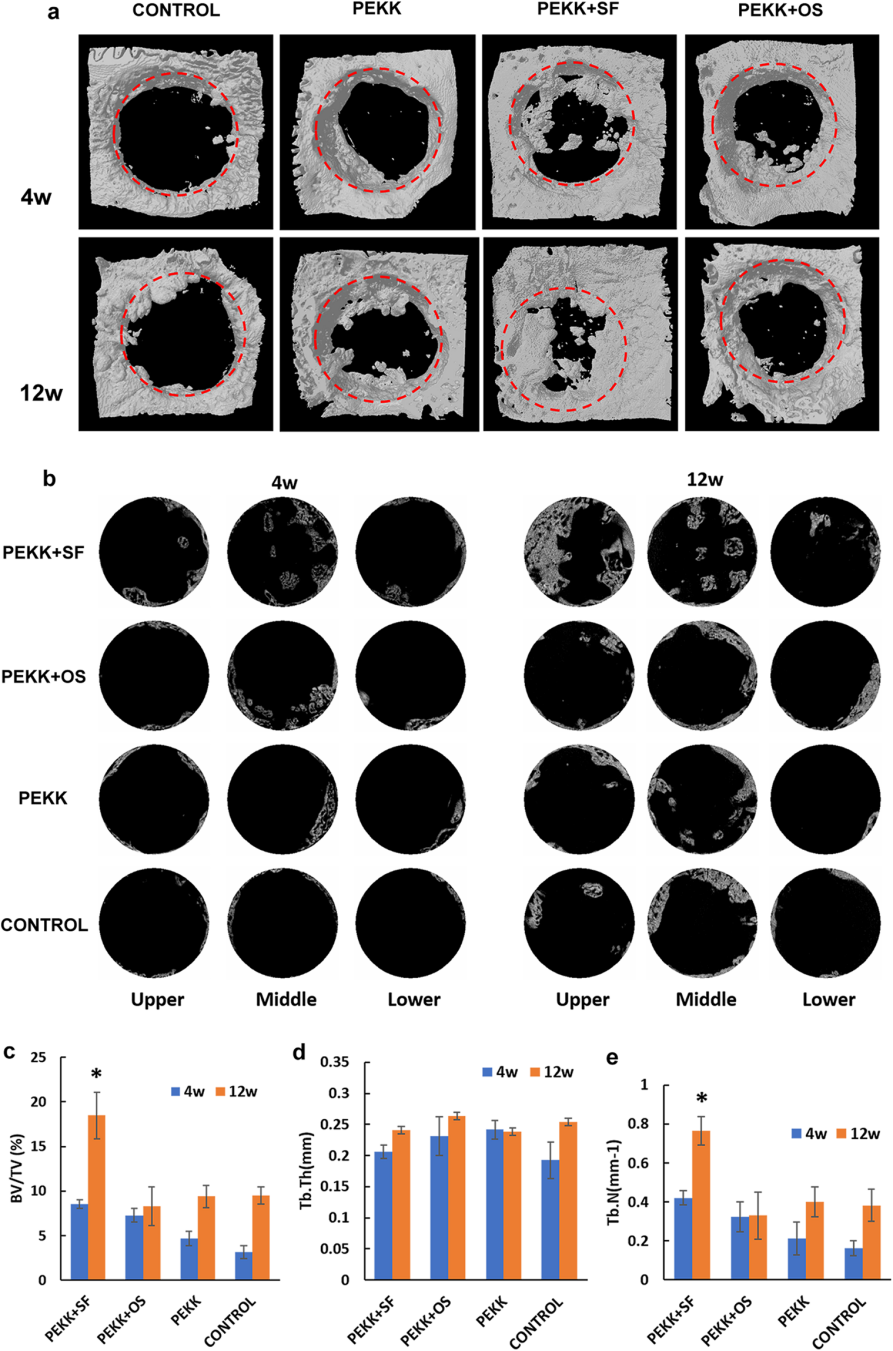
Micro-CT analysis of grafts in the critical-sized defect area. (**a**) 3D reconstruction of newly formed bone within the critical-sized defect after 4-week and 12-week with no graft (Control), PEKK, PEKK + SF, and PEKK + OS, respectively. The red dotted circles outlined the margin of the original 8-mm defect. (**b**) Micro-CT images of the trabecular structures seen at three different depths within the critical-sized defects (upper, middle, and lower layers at 450 µm distance from each other) at 4 weeks and 12 weeks post-implantation. (**c–e**) Quantitative analysis of newly formed bone in the critical-size defect at 4 weeks and 12 weeks for each implant group. Note: BV/TV: the percentage of bone volume to total volume of the defect; Tb.Th: trabecular thickness; Tb.N: trabecular numbers. Data are presented as mean ± SE. *p < 0.05.

Using micro-computed tomography (micro-CT) and 3D image reconstruction, the regenerated bone within the critical-sized defects could be measured. The PEKK material/implant was radiolucent and could be discriminated from the newly-formed bone. Micro-CT analysis revealed that bone regeneration occurred in all four groups at 4 and 12 weeks post-surgery, but at different rates (Fig. 4g,h). Detailed and quantitative bone growth measurements were shown in Fig. 5. Each original critical-sized defect margin was reconstructed using a hypothetical red-dotted circle of 8 mm in diameter (Fig. 5a). Bone regeneration started at the margin of the surgically-created osseous defect and gradually moved toward the center of the defect. Fig. 5b showed cross-sectional images at three different depths of the defects (8 mm diameter by 1.5 mm height). These images demonstrated that new bone regenerated faster at the top (superficial) of the defect than at the bottom of the defects. Defects implanted with hSF-MSCs seeded on PEKK scaffolds (PEKK + SF) had the highest volume of regenerated bone at 12 weeks (Fig. 5a,b). These observations were supported by measurements of bone volume normalized to the total volume of the defect (BV/TV) indicating that, at 12 weeks, hSF-MSCs seeded on PEKK (group PEKK + SF) had ~20% bone volume versus 9–10% for the other 3 groups (Fig. 5c; *p* < 0.05). In terms of trabecular structure, the cross-sectional images of the upper, middle, and lower portions/depths of the defects showed that bone grew in an organized pattern around the connecting pores of the scaffolds for the PEKK + SF group (Fig. 5b). The distribution of the regenerated bone in the PEKK + SF group was relatively uniform across the scaffold depth. No significant differences in trabecular thickness (Tb.Th) were found among the four groups (Fig. 5d). However, trabecular numbers (Tb.N) in the PEKK + SF group was higher than the other groups (0.80 ± 0.05 mm^−1^, *p* < 0.05) (Fig. 5e). This meant that PEKK scaffolds with hSF-MSCs regenerated more bone tissue *in vivo* than scaffolds without seeded cells. Additionally, hSF-MSCs provided more bone regeneration than osteogenically-induced hSF-MSCs (PEKK + OS group).

**Figure 6:**
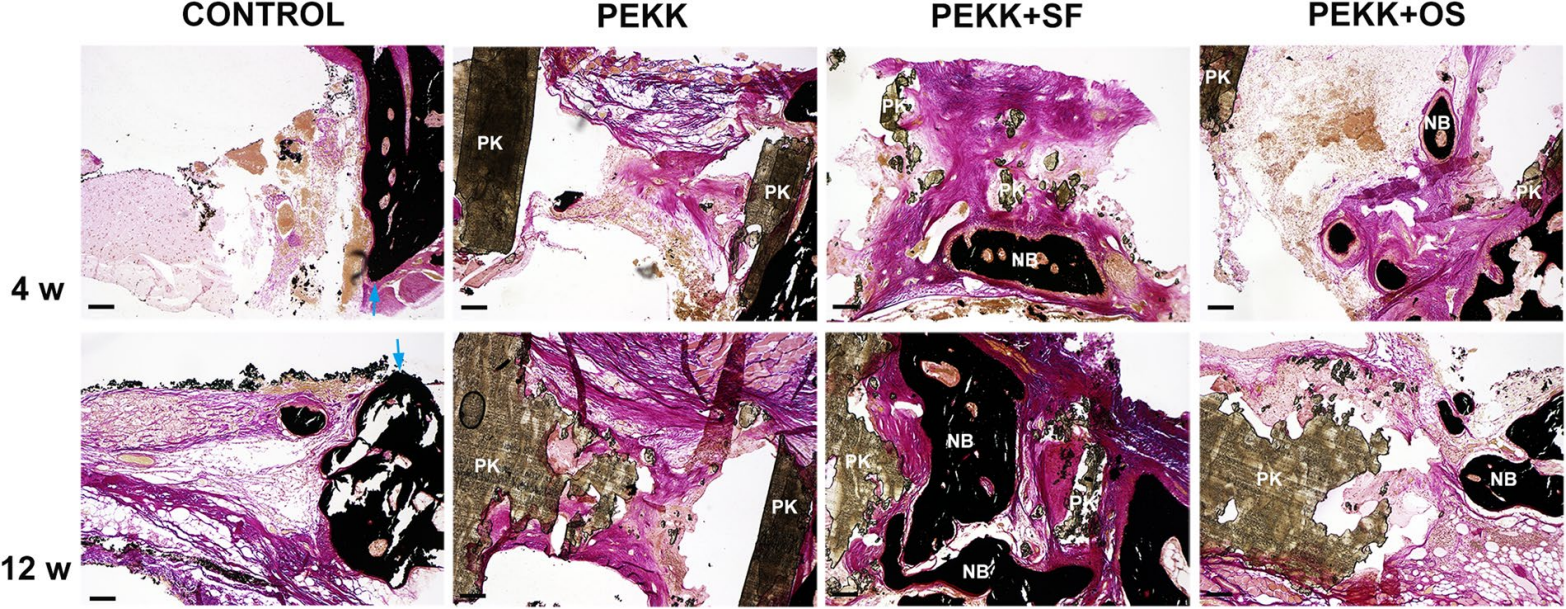
Representative histological sections stained with von Kossa/van Gieson at 4 and 12 weeks. Mineralized bone is stained in black. Blue arrowheads indicate the edges of old bone; NB: new bone; PK: PEKK; Scale Bar: 150 µm.

Histological staining of the explanted calvarial specimens was performed to examine the mineralization status of the newly formed bone in the critical-sized defects (Fig. 6). Four weeks and 12 weeks post-surgery, von Kossa staining identified very little mineralization in defects without scaffolds (Control group) or with cell-free scaffolds (PEKK group). Conversely, a considerable amount of mineralized bone tissue was found between the microchannels of PEKK seeded with hSF-MSCs (PEKK + SF group). Bone marrow-like tissue was observed within the new bone. Larger amounts of newly formed mineralized bone were observed at 12 weeks when compared to 4 weeks. There was relatively less new bone formed in the PEKK + OS group, and the newly-formed bone was located at the periphery of the defect.

**Figure 7:**
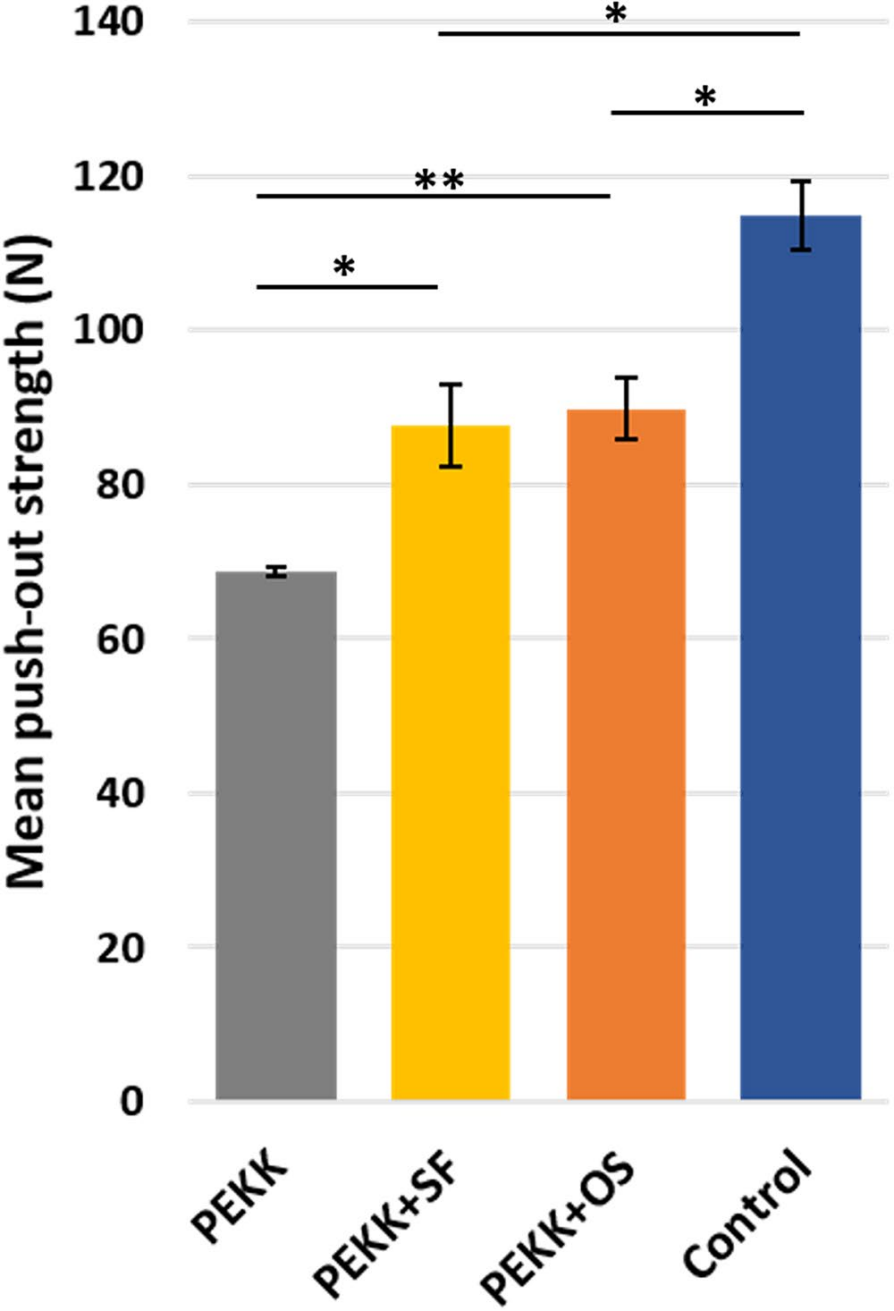
The mechanical push-out strength of the calvarial regenerated bone. Native calvaria had the highest push-out strength value, followed by PEKK + SF and PEKK + OS, which is significantly higher than PEKK grafts. No significant difference was measured in the push-out strength of PEKK seeded with hSF-MSCs with or without osteogenic-induction. Data are presented as mean ± SE. *p < 0.05 and **p < 0.01.

The mechanical push-out test was used to measure the functional strength of the regenerated bone. It was observed that PEKK + SF (87.6 ± 5.4 N, *p* < 0.05) and PEKK + OS (89.9 ± 4.0 N, *p* < 0.05) groups had significantly greater push-out strengths than the PEKK group (Fig. 7). No statistical difference was found between PEKK + SF and PEKK + OS. The positive control group was the native intact calvarial bone (114.9 ± 4.4 N), while the lowest strength was observed in PEKK group without cells (68.7 ± 0.7 N).

## Discussion

Cells used in this study were isolated from human synovial fluid of TMJ from TMD patients. In our pilot studies, hSF-MSCs of healthy volunteers were obtained and isolated, but these were not used in the current study. Those MSCs from healthy donors shared similar flow cytometric characteristics to MSCs from TMD patients. However, the main difference was that hSF-MSCs could only be isolated from ~16.7% of healthy donors TMJ synovial fluid^45^. Also, the number of colony forming units (CFUs) of hSF-MSCs from healthy donors at plating (passage 0) was much lower, and this resulted in an insufficient cell number for our subsequent experiments. Another reason for using hSF-MSCs from TMD patients was the availability of the synovial fluid following the arthrocentesis treatment; while synovial fluid collection would not have been indicated for healthy patients (i.e. without TMD). Furthermore, our long-term goal is to use autologous hSF-MSCs to reconstruct the mandibular condyle of patients suffering from severe TMJ osteoarthritis, and thus this was another reason for selecting hSF-MSCs from TMD patients for this study. hSF-MSCs displayed typical MSCs characteristics based on their cell surface markers (CD73, CD90, CD105, CD45, etc), multipotency, and self-renewal capacity (Fig. 1). In our previous study about hSF-MSCs^34,46–48^, Flow cytometric experiments were done from the third to sixth passages of hSF-MSCs. At passage 6^th^, more than 96% of hSF-MSCs were positive for MSC markers and less than 2% of hSF-MSCs were positive for (CD45, CD34, CD11b, CD19, and HLA-DR). These flow cytometric results were not statistically different when compared to those cells at passages 3^rd^ to 5^th^. CD44 is a receptor for hyaluronic acid, which is a major component of articular surfaces^49^. Previous reports focused on the chondrogenesis function of hSF-MSCs and their potential applications in cartilage repair^24,26,35^. hSF-MSCs were also reported to have comparable biological characteristics with BMSCs and possessed a greater osteogenic potential than other MSCs obtained by non-invasive procedures, such as the dental pulp or exfoliated deciduous teeth stem cells^23,26^. However, their contributions in regenerating bone defects have barely been investigated in the literature. By testing the osteogenic ability of hSF-MSCs on 3D-printed scaffolds both *in vitro* and *in vivo*, our study proposed an additional source of non-invasively harvested human MSCs for bone regeneration as well as an optimal source of autologous cells to repair bone defects of TMD patients.

The first finding of this study was that the surface of 3D-printed PEKK scaffolds was biocompatible for cell attachment and growth. PEKK belongs to the polyaryletherketones family (PAEKs) which are materials with mechanical properties that can coexist with human bone^37^. PEKK is considered a promising material to replace metals and ceramics currently used in orthopedics^36,38,50^ and in dentistry^42,43,51–53^. However, there are only a few studies^36,39^ reporting the effect of PEKK on stem cells. This study tested a 3D-printed PEKK scaffold with 750–1000 µm interconnecting channels. The laser sintering process resulted in a rough and porous surface, when observed at the SEM level (Fig. 2a,d). This surface topology might have favored cell attachment and their osteogenic differentiation. Although hSF-MSCs proliferated slower on PEKK than on tissue culture plastic (TCP; 2D culture), cells on PEKK showed multiple filopodia and lamellipodia (Fig. 2b,e,f). Both structures are composed of actin fibers and transmembrane adhesion complexes. Filopodia act as sensors to explore the extracellular environment whereas lamellipodia can induce cell migration by supporting traction forces from the cell actomyosin network^54,55^. These images (Fig. 2b,e,f) suggested that the surface of our 3D-printed PEKK had adhesive interactions with the seeded cells and might modulate cellular signalings toward osteogenesis.

The second finding of this study was that the PEKK scaffold by itself could not induce osteogenesis in hSF-MSCs but required the osteogenic cultured media. As expected, hSF-MSCs cultured on either PEKK or TCP, and in non-osteogenic media did not show an osteogenic differentiation. However, when hSF-MSCs were cultured in osteogenic media, this cultured condition enhanced the osteogenic differentiation of hSF-MSCs as the result shown in groups PEKK + OS and TCP + OS (Fig. 3). ALP activity levels were higher in hSF-MSCs grown on PEKK than on TCP, as early as day 4 and day 7 (Fig. 3a). Also, an upregulation of osteogenesis-related genes was detected at day 21 for cells grown on PEKK (Fig. 3b). ALP is a transient early marker of osteogenic differentiation for MSCs^56^, indicating their ability to differentiate into osteoblasts^57^. An initial rise in ALP activity, with a peak at day 14, was observed in PEKK + OS and TCP + OS groups, which was in line with several previous studies^57–59^. This observation was confirmed with the downregulation of the *ALP* gene at day 21. Unlike *ALP*, other osteogenic genes such as *OPN*, *OCN*, *COLІA1*, and *RUNX2*, all exhibited a significant upregulation when cultured in osteogenic media for 21 days. These markers were commonly used in many other studies to demonstrate the osteogenic potential of MSCs, and whether the MSCs were at their early or late stage of osteogenic differentiation. To obtain an overall ability of hSF-MSCs in maintaining their osteogenic phenotypes when cultured on PEKK scaffolds, the mRNA levels of these markers were measured at day 21, which was a widely accepted timepoint in the literatures. *RUNX2* encodes a key transcription factor with an essential role in the commitment of MSCs towards osteoblasts^60^. However, its expression pattern during osteogenesis varies. Huang X *et al*.^61^ reported that the pattern of *RUNX2* expression fluctuated during osteogenic differentiation of MSCs. The expression of *RUNX2* was found to increase initially and then to decrease in human dental pulp MSCs, while it remained consistently high in human bone marrow MSCs during their osteogenic/odontogenic differentiation^61^. It was also reported that the mRNA level of *RUNX2* correlate positively with the level of *OCN* during osteogenesis^62^. Therefore, we measured both the osteogenic late markers and their upstream regulator (*RUNX2*) to allow us to detect their correlations. The mRNA level of *RUNX2* was significantly higher in the PEKK + OS group. Not surprisingly, the expression of *OCN* on PEKK was 5.8 folds higher than the TCP + OS group, suggesting an enhanced mineralization of the extracellular matrix (ECM) and more active osteoblasts. ECM provides a template for mineralization, which mainly consists of collagens (especially collagen type І)^63^. A higher expression of *COLIA1* indicated a more active formation of ECM on PEKK relative to TCP. The highest upregulated gene that we measured during hSF-MSCs culture was *OPN*. This indicated a proliferation of pre-osteoblasts *in vitro* as OPN is secreted prior to matrix mineralization^64^. The increased gene expression levels of *OPN*, *OCN*, *COLІA1*, and *RUNX2* implied that hSF-MSCs cultured on PEKK 3D-scaffolds were induced into an accelerated osteogenic differentiation *in vitro*. Such benefits of PEKK (i.e. increased mRNA levels toward osteogenesis) along with its biocompatibility and biomechanical properties confirmed its use as an adequate scaffold for bone tissue engineering.

The third finding of this study was that (naïve/non-differentiated) hSF-MSCs were more effective in regenerating bone when compared to osteogenically-induced hSF-MSCs. Our *in vivo* results demonstrated that the volume of bone formed (~20%) by hSF-MSCs was twice higher than the bone volume (~10%) of osteogenically-induced hSF-MSCs. This was a surprise to us because some researchers had previously reported that it was better to osteogenically-induced MSCs prior to their *in vivo* transplantation to increase bone regeneration^17,65^. We hypothesized that the undifferentiated/naïve hSF-MSCs possessed a greater cell plasticity and responded to the rich cocktail of healing stimulus in the critical-sized defects^16,17,66^. Nevertheless, the decision to transplant naïve/non-differentiated versus osteogenically-induced MSCs to increase bone formation remained controversial^16,17,65,67^. This discrepancy might be due to studies using MSCs from different tissues, different scaffold materials, and different animal models. Results from this study indicated that there was no need to osteogenically-induced human MSCs from TMJ synovial fluid when cultured on PEKK prior to their *in-vivo* implantation into the rabbit cranial critical-sized defect model.

Last but not the least, our *in-vivo* results supported the use of PEKK as a scaffold for hSF-MSCs. Our micro-CT and histological results (Figs 5 and 6) revealed a distinct increase in bone regeneration when either hSF-MSCs (PEKK + SF) or osteogenically-induced hSF-MSCs (PEKK + OS) were seeded on PEKK scaffolds. According to our histological analysis, non-differentiated hSF-MSCs (from treatment group PEKK + SF) formed larger and more matured osteoid, while osteogenically-induced hSF-MSCs (from treatment group PEKK + OS) produced fewer and smaller mineralized tissue, which was mainly located to the periphery/border of the critical-sized defect (Fig. 6). However, newly-formed peripheral bone still contributed to the integration of PEKK into the critical-sized defect because results from the Push-Out Strength test of both PEKK + SF and PEKK + OS groups were higher than those from the PEKK group (that had no seeded hSF-MSCs) (Fig. 7). Histologically, we did not detect an increased number of inflammatory cells when PEKK was transplanted with hSF-MSCs. This result suggested no (observable) immunological rejection of the xenogeneic (human) hSF-MSCs in the rabbit model. Our observations were in agreement with earlier studies supporting the immunomodulatory properties of human MSCs^68^. Still, the rabbit calvarial site might be more immune-privileged^69^ and we could not exclude this possibility. On the contrary, Adamzyk *et al*.^36^ found no added effect of transplanted ovine BMSCs on bone regeneration in sheep calvarial defects. Adamzyk and colleagues reported, in their flow cytometric data, a lower percentage of MSCs from ovine bone marrow. Also, the sheep model mounted an immunological response to transplanted allogeneic cells^36^. These two factors might explain a decreased potential of bone regeneration observed by these authors.

Although the bone defect was not completely healed within the 12-week duration of this study, the regenerated bone in the group that was implanted with PEKK seeded with hSF-MSCs demonstrated a functional recovery. Additional studies with a longer follow-up period are required to investigate whether complete closure of the bone defect would be established with our proposed grafting material combined with hSF-MSCs. Also, to further confirm the benefits of PEKK scaffolds, additional studies comparing PEKK to other typical bone scaffolds will be needed.

## Conclusions

In conclusion, hSF-MSCs from the TMJ could be easily harvested with minimal invasiveness to the patients. hSF-MSCs possessed high multipotency and could be an alternate cell source to BMSCs for bone regeneration, as well as an optimal autologous cell source for TMJ repair. The combination of hSF-MSCs and PEKK (a biocompatible bone-mimetic polymer) resulted in an increased osteogenic ability, both *in vitro* and *in vivo*. The combined implantation of PEKK and hSF-MSCs is a promising therapy to regenerate large bone defects.

## Methods

### PEKK Scaffold fabrication

PEKK scaffolds were designed using SOLIDWORKS® (version 2015, Vélizy-Villacoublay, France). The porosity was set at approximately 60% with a strut size of 1.15 mm, resulting in a printed pore size of 750–1000 μm. With an EOSINT P 800 printer (EOS GmbH, Germany), the PEKK scaffolds were printed using a laser sintering technique (Oxford Performance Materials, CT, USA) and then cut into cylinders (8 mm diameter and 1.5 mm thickness).

### Isolation and culture of hSF-MSCs

hSF-MSCs were isolated from the synovial fluid aspirated from the TMJ cavity of TMD patients undergoing arthrocentesis^34^. The synovial fluid sample was centrifuged at 300 g for 5 min and cells were resuspended and cultured in standard culture media, which is complete alpha-minimum essential medium (α-MEM; Gibco BRL, NY, USA) supplemented with 10% fetal bovine serum (FBS; Gibco) and 1% penicillin/streptomycin (Gibco), at 37 °C in 5% humidified CO_2_. The cultured media was replaced every 2–3 days until 80% cell confluence. Cells were then detached with 0.25% trypsin EDTA (Gibco), resuspended in standard culture media, and replated at a density of 5000 cells/cm^2^ for expansion. Cells used for experiments in this study were from the 3^rd^ to 5^th^ passages.

### Characterization of hSF-MSCs

hSF-MSCs were then cultured in specialized media to evaluate their adipogenic, osteogenic, or chondrogenic characteristics. Adipogenesis was induced by α-MEM supplemented by 15% FBS, 0.5 mM isobutylmethylxanthin, 0.01 µM dexamethasone sodium phosphate (Sigma), 60 µM indomethacin, 0.5 µM hydrocortisone, 10 µg/mL insulin, 55 µM beta-Mercaptoethanol and 0.1 mM L-ascorbic acid phosphate for 14 days. Osteogenesis was induced by osteogenic media containing α-MEM containing 15% FBS, 0.01 µM dexamethasone sodium phosphate (Sigma), 0.1 mM L-ascorbic acid phosphate, and 2 mM beta-glycerophosphate for 21 days. Chondrogenesis was induced using the Mesenchymal Stem Cell Identification Kit (R&D system, MN, USA) for cells pelleted in a 15 mL tube for 28 days. Subsequently, cells were stained by Oil Red O (Sigma), Alizarin Red (Sigma), and anti-collagen type II antibody (Abcam, MA, USA), respectively. Other sets of hSF-MSCs were refreshed by standard culture media as controls.

For flow cytometric analysis, cells were suspended in PBS before being incubated with the following antibodies (BD Biosciences, CA, USA) for one hour at room temperature: Fluorescein isothiocyanate (FITC)-conjugated anti-human CD44, CD90, CD105, CD73, CD45, CD34, CD11b, CD19, and HLA-DR. The phenotype analysis was then performed using an FC 500 flow cytometer (Beckman Coulter).

### Cell Proliferation on scaffolds

The PEKK scaffolds were sterilized by high-pressure steam (Yamato SM 300, Japan). The scaffolds were seeded with hSF-MSCs at a cell concentration of 5.0 × 10^4^ cells/mL. hSF-MSCs were also seeded, using the same concentration and volume, on tissue culture plastic (TCP) as controls. Cell proliferation was evaluated at 1, 3, 5, and 7 days by the Alamar Blue assay (Invitrogen, Carlsbad, CA). Cell/scaffold complex were incubated in 0.5 mL of complete culture medium with 10% (v/v) Alamar blue solution for 3 h. The fluorescence intensity of the culture media at 570/585 nm was measured in triplicates using a microplate reader (SpectraMax^®^ M2, USA).

### SEM

After culturing hSF-MSCs on PEKK for 7 days, the cell/scaffold complexes were collected, washed three times with PBS, and fixed in 2.5% (v/v) glutaraldehyde for at least 24 h at 4 °C. Then, the samples were rinsed three times, followed by serial dehydration in 30%, 50%, 70%, 90%, and 100% ethanol. Hexamethyldisilazane was used before the samples were air-dried and sputtered with gold (Leica EM ACE600, Wetzlar, Germany). The surface morphology was analyzed by FEG Quanta Scanning Electron Microscope (Inspect F50, FEI Company, OR, USA) at 5 kV to image.

### ALP activity

Cell suspension, at a density of 2 × 10^5^ cells/mL, was evenly added onto PEKK/TCP dropwise. Four groups were tested *in vitro*: (1) PEKK + OS (PEKK seeded with hSF-MSCs in osteogenic media), (2) PEKK + SF (PEKK seeded with hSF-MSCs in standard culture media), (3) TCP + OS (TCP seeded with hSF-MSCs in osteogenic media), and (4) TCP + SF (TCP seeded with hSF-MSCs in standard culture media). After 3 days of culture, the standard culture media in the OS groups was changed into an osteogenic media and renewed twice a week while the SF groups were renewed with standard medium at the same frequency. The cellular ALP activity was detected after 1, 4, 7, 14, and 21 days^70,71^. Sigma*Fast* p-nitrophenyl phosphate tablets (Sigma) dissolved in deionized H_2_O was used with the cell lysate to detect the ALP level. The absorbance was read in a microplate reader at 405 nm (SpectraMax^®^ M2). The ALP units were standardized to the cell number.

### qRT-PCR

The expression levels of osteogenic-related genes *OPN*, *OCN*, *ALP*, *COLIA1*, and *RUNX2* were measured using qRT-PCR after osteogenic induction for 21 days. Total RNA was extracted from hSF-MSCs with TRIZOL (Invitrogen). 50 ng RNA per sample was reverse-transcribed into the first strand cDNA synthesis with Thermoscript RT-PCR system (Invitrogen). The sequence of the primers used in this study is listed in Table 1. Triplicate qRT-PCR assays were performed using Step One Plus (Life Technologies, USA) in TaqMan Universal Master Mix II (Applied Biosystem, Foster City, Canada). The relative expression of the genes of interest was normalized against the housekeeping gene *GAPDH*. Triplicate experiments were conducted for each sample.

**Table 1:** The sequence of primer.

Gene name	Abbreviation	Assay ID	Amplicon length
Osteopontin	*OPN*	Hs00959010_m1	84
Osteocalcin	*OCN*	Hs01587814_g1	138
Alkaline phosphatase	*ALP*	Hs01029144_m1	79
Collagen type I alpha 1	*COLIA1*	Hs00164004_m1	66
Runt-related transcription factor 2	*RUNX2*	Hs01047973_m1	86
Glyceraldehyde-3-phosphate dehydrogenase	*GAPDH*	Hs99999905_m1	122

### Animal Surgery

The animal study was performed according to regulations of the Canadian Council on Animal Care and the protocol was approved by McGill ethics committee (AUP2015–7571). Four critical-sized defects were created in the calvarial bone of female New Zealand White rabbits weighing between 2.5–3.0 kg (N = 10). The critical-sized cranial defects in each rabbit were randomly allocated to four treatment groups: (1) PEKK + SF (PEKK seeded with hSF-MSCs precultured in standard culture media; (2) PEKK + OS (PEKK seeded with hSF-MSCs precultured in osteogenic media for 14 days; these hSF-MSCs were referred as “osteogenically-induced hSF-MSCs” throughout this study; (3) PEKK (PEKK alone); and (4) Control (The defect was left empty).

For anesthesia, acepromazine (0.75 mg/kg), xylazine (5 mg/kg), and ketamine (20–35 mg/kg) were injected intramuscularly followed by isoflurane (1.5–3%) delivered in 100% oxygen (flow rate 0.8–1.5 L/min for induction and 0.4–0.8 L/min for maintenance) through endotracheal intubation inhalation^72^. Ancef (12 mg/kg) was given intramuscularly prior to incision. A 4-cm long skin incision over the linea media was made, and the tissue was dissected to adequately expose the calvaria. Using an electric hand piece with a trephine bur, four circular bicortical defects (8 mm in diameter) were created symmetrically to the midline of the calvaria in the parietal bones under constant saline irrigation (Fig. 4a,b). After the transplantation procedure (Fig. 4c), the layers were closed separately with resorbable 4-0 sutures. Baseline computed tomography images (at day 0) of the rabbits were taken immediately after the surgery (Fig. 4d). Buprenorphine (0.05 mg/kg) was administered for 48 h to reduce post-operative pain. Rabbits were sacrificed either at the 4th (Fig. 4e) or 12th week (Fig. 4f) postoperatively, and their calvaria were removed for further analyses.

### Micro-CT analysis

The explanted specimens were fixed with 10% buffered formalin and then stored in 70% ethanol. Micro-CT was performed to quantify the volume of bone formation within the defects using the Skyscan 1072 scanner (Bruker-MicroCT, Kontich, Belgium) at 55 kV, 181 µA, and a spatial resolution of 15.96 μm pixel through an Aluminum filter^72^. The micro-CT data were reconstructed by NRecon software (Bruker-MicroCT), realigned to the primary axes of the cylindrical defect space by DataViewer (Bruker-MicroCT), and the 3D image stack was analyzed by CTAn (v1.13; Materialise, Leuven, Belgium). A cylindrical shape of 8 mm in diameter and 1.5 mm in height was selected at the center of the bone tunnel/defect as the volume of interest. The same threshold was used among all groups. Bone volume and trabecular pattern were analyzed afterwards, followed by 3D visualization using CTVol (v13.0; Materialise).

### Histology

Samples were dehydrated through a series of ethanol concentrations and then embedded in methyl methacrylate. Thin undecalcified tissue slices (7 µm) were cut perpendicularly to the bone surface using a rotary microtome (Leica RM2255, Germany). These slices were stained with von Kossa and counterstained with van Gieson to distinguish the mineralized non-mineralized tissues.

### Push-out test

At week 12 post-operatively, the explanted specimens were stored in cold PBS and tested by a push-out machine (TestResources 313, Shakopee, USA). Parietal bone samples without surgical defects were used as positive controls. The specimens were placed on a customized fixed rigid platform with a 9 mm hole and a 6 mm flat indenter centered in the defect area. The surface of the defect was aligned parallell to the surface of the indenter. The force and displacement were recorded when the indenter moved into the repair area at a speed of 5 mm/min until the specimen failed/broke. XY Software (Version 4.00.10, Shakopee) was used to analyze the data and identify the peak compressive force for each set of data.

### Statistical analysis

All data were analyzed using SPSS 19.0 software. Statistical analysis was conducted using one-way analysis of variance followed by the Bonferroni test for multiple comparisons among the experimental groups. These data were presented as mean ± standard error of the mean (SE). Value of *p* < 0.05 were considered significant.

### Ethics approval and consent to participate

Human synovial samples were collected after informed consent was provided in accordance with the Declaration of Helsinki and under the approval of the Ethics Committee for clinical research at the Hospital of Stomatology, Guanghua School of Stomatology, Sun Yat-sen University, China (ERC-2016-18). All procedures with live animals were performed in accordance with regulations set by the Canadian Council on Animal Care and the protocol was approved by McGill ethics committee (Protocol number: AUP2015–7571).

## Data Availability

The datasets generated during and/or analyzed during the current study are available from the corresponding authors.
